# Transmitted drug resistance associated with transmission clusters in newly diagnosed antiretroviral-naïve patients in Northern Greece

**DOI:** 10.1186/1758-2652-13-S4-P125

**Published:** 2010-11-08

**Authors:** L Skoura, S Metallidis, AJ Buckton, JL Mbisa, D Pilalas, E Papadimitriou, A Papoutsi, AB Haidich, D Valagouti, O Tsachouridou, ZA Antoniadou, P Kollaras, P Nikolaidis, N Malisiovas

**Affiliations:** 1 Medical School, Aristotle University of Thessaloniki, National AIDS Reference Center of Northern Greece, Thessaloniki, Greece; 2 AHEPA University Hospital, Medical School, Aristotle University of Thessaloniki, Infectious Diseases Division, Thessaloniki, Greece; 3 Health Protection Agency, Colindale, Virus Reference Department, Centre for Infections, London, UK; 4 Medical School, Aristotle University of Thessaloniki, Department of Hygiene, Thessaloniki, Greece

## Purpose of the study

To determine the contribution of transmission clusters on transmitted drug resistance (TDR) in newly diagnosed antiretroviral naive patients in Northern Greece, during 2000 -2007.

## Methods

Viral reverse transcriptase and protease genes from 369 individuals with newly diagnosed HIV-1 infection were sequenced at baseline. A maximum-likelihood phylogenetic analysis method was employed to examine for linkage between viral isolates. Clinical data were retrieved from the database and cross-referenced with the patients' medical files. Transmitted drug resistance was defined in accordance with the Surveillance Drug Resistance Mutation (SDRM) 2009 list.

## Results

The study population characteristics were as follows: 82.8% male, 89.7% of Greek nationality, mean age 38, median CD4 cell count at diagnosis 295 cells/µl and mean HIV-1 RNA 4.94 log10 copies/ml. The most prevalent risk exposure category was men who have sex with men 59.1% (n=218) followed by heterosexual transmission 21.4% (n=79) and intravenous drug use 7.6% (n=28). Subtype B viruses were most prevalent (53.1%), followed by subtype A (32.5%). At least one drug resistance mutation was identified in 46/369 patients (12.4%). Twenty-eight patients (7.6%) harbored resistance mutations to nucleoside/nucleotide RT inhibitors, 20 patients (5.4%) to non-nucleoside RT inhibitors and 12 patients (3.3%) to protease inhibitors (PIs). Dual-class resistance mutations were identified in 14 patients (3.8%). The median CD4 cell count in patients with TDR was not significantly different compared to patients without (p=0.072). Phylogenetic analyses, supported by bootstrapping >90% and genetic distance <0.015, revealed three transmission clusters involving drug resistant strains, including one cluster of 11 patients infected with a strain carrying RT mutations Y181C and T215 variants conferring NRTI and NNRTI resistance.

## Conclusions

The overall prevalence of TDR in our study population was 12.4%. Phylogenetic analyses of viral sequences from these new diagnoses demonstrated the impact of transmission clusters on the primary drug resistance. The outbreak of dual-class TDR, coupled with late HIV diagnosis in this population may require improved public health interventions.
